# Influence of heart rate trajectory in 30-day mortality in sepsis patients: a retrospective study based on the MIMIC-IV database

**DOI:** 10.1186/s12879-025-11961-9

**Published:** 2025-12-09

**Authors:** Wenming Shao, Jianlong Yan, Yanbin Pan, Yabin Jiang, Hui Liu, Luming Zhang, Jun Lyu, Haiyan Yin

**Affiliations:** 1 Emergency of Department, The First Affiliated Hospital of Jinan University, 613 Huangpu Avenue West, Guangzhou City, Guangdong Province China; 2 Department of Intensive Care Unit, The First Affiliated Hospital of Jinan University, Guangzhou, China; 3 Department of Clinical Research, The First Affiliated Hospital of Jinan University, Guangzhou, China; 4 Department of Health Management Center, Shenzhen People’s Hospital (The Second Clinical Medical College, Jinan University, The First Affiliated Hospital, Southern University of Science and Technology), Shenzhen, Guangdong China; 5 Department of Cardiology, Shenzhen Cardiovascular Minimally Invasive Medical Engineering Technology Research and Development Center, The Second Clinical Medical College, The First Affiliated Hospital, Shenzhen People’s Hospital, Jinan University, Southern University of Science and Technology), Shenzhen, Guangdong China

**Keywords:** Sepsis, Heart rate, Trajectory, Latent growth mixture modeling

## Abstract

**Background:**

Heart rate is one of the important vital signs. Many studies have done a lot of meaningful research on heart rate in sepsis patients. However, the heart rates of sepsis patients were repeatedly measured after their admission to intensive care unit (ICU). The trajectory of these changes was observed, but the impact of this trajectory on the short-term mortality prognosis for sepsis remains unclear. This study was performed to investigate impact of repeated changes in heart rate on short-term all-cause mortality among sepsis patients was assessed.

**Method:**

In this retrospective study of data on sepsis patients from the Medical Information Mart for Intensive Care (MIMIC) IV database, the outcome was short-term mortality. The measured heart rate of sepsis patients 10 h post-admission to ICU was extracted, with 1 h between each measurement. Latent growth mixture modelling (LGMM) was used to classify heart rate trajectories, while Kaplan–Meier (K-M) and Cox proportional hazards models were employed to analyze differences in survival between groups.

**Result:**

This study enrolled 5511 sepsis patients, and six different heart rate trajectories were identified based on model-fit criteria. Class 1: heart rate stable at 80 bpm; class 2: consistently 92 bpm; class 3: briefly stable, then declining; class 4: steady at 110 bpm; class 5: fluctuating around 65 bpm; class 6: stable at 128 bpm. The K-M analysis indicated sepsis patients in class1 had the highest survival probability, and the class3 had the lowest survival probability. After adjusting for all potential confounding factors, the Cox proportional hazard model showed that compared with class 1, the hazard ratios (HRs) with 95% confidence intervals (CIs) for classes 2, 3, 4, 5, and 6 were 1.28 (1.09, 1.50), 1.88 (1.38, 2.56), 1.49 (1.26, 1.76), 1.24 (1.03, 1.48), and 1.30 (1.02, 1.66), respectively.

**Conclusions:**

Maintaining a sustained heart rate of approximately 80 beats per minute during the initial 10-hour period post-ICU admission may be associated with improved short-term outcomes in patients with sepsis.

**Supplementary Information:**

The online version contains supplementary material available at 10.1186/s12879-025-11961-9.

## Introduction

 Sepsis is a life-threatening condition resulting from the body’s abnormal response to infection, which can lead to organ dysfunction. Studies have shown that it affects up to 437 out of every 100,000 people in developed countries [1], with infection-related deaths accounting for 54% of all deaths worldwide [2]. It is also one of the most common causes of death among patients in intensive care units (ICUs) [3]. Sepsis imposes a huge burden on global healthcare resources and economy [4], and is a serious threat to human life and health [5]. It is imperative to identify the early risk factors of sepsis and intervene promptly to halt its progression. Previous studies have established that an elevated resting heart rate is an independent risk factor for both the general population and those with cardiovascular diseases [6]. Among septic patients and those studied with Multiple Organ Dysfunction Syndrome (MODS), it has been observed that those whose heart rates decreased within 24 h of ICU admission had a higher survival rate. Heart rate is governed by both the autonomic nervous system and the sinoatrial node. During sepsis, significant alterations in these regulators can lead to tachycardia [7]. Although current research suggests that maintaining a relatively lower heart rate may be beneficial for sepsis patients, the optimal range for such control remains a subject of debate. It is widely recognized in clinical settings that dynamically monitoring patients’ clinical indicators, detecting issues promptly, and intervening early holds greater clinical significance than relying on singular measurements [8]. According to current literature, the trajectory of heart rate development in sepsis patients and its impact on short-term prognosis remains nebulous.

The objective of this study is to elucidate the influence of heart rate progression on the short-term prognosis of sepsis patients by employing the Latent Growth Mixture Modeling (LGMM). This endeavor seeks to identify the optimal prognostic heart rate category measurement as a benchmark for expected benefits, thereby mitigating the short-term mortality risk in sepsis patients.

## Methods

### Ethics approval and consent to participate

The study was an analysis of a third-party anonymized publicly available database with pre-existing institutional review board (IRB) approval. Data extracted from the MIMIC-IV database do not require individual informed consent because MIMIC-IV database research data is publicly available and all patient data are de-identified.

### Data source

The source of the data for this retrospective was retrieved from the Medical Information Mart for Intensive Care (MIMIC-IV 2.0), which comprises clinical data from a custom hospital-wide electronic health record and an ICU specific clinical information system for more than 380,000 patients who were admitted to the Beth Israel Deaconess Medical Center (BIDMC) in Boston, Massachusetts, USA, between 2008 and 2019. These records include the diagnosis, vital signs, laboratory tests, medication, and surgical information, ect [9, 10]. To protect patient privacy, all private information in the database depository has been removed. Thus, informed consent and the ethical approval statement were waived for this study. Researchers need to complete the corresponding courses required by the database and pass the assessment in order to access and obtain the data of the database.

### Data extraction

This study drew upon data from patients identified with sepsis in alignment with the Sepsis 3.0 guidelines [11]. These guidelines necessitate a diagnosis of either a suspected or confirmed infection paired with a Sequential Organ Failure Assessment (SOFA) score of ≥ 2. The exclusion criteria were as follows: (1) age < 18years or >90 years, (2) ICU stay less than 48 h, (3) sepsis patients whose heart rate wasn’t monitored for a consecutive 10-hour duration, with hourly measurements, (4) multiple ICU stays. Data extraction was conducted from the MIMIC-IV database using Structured Query Language (SQL).

### Variables and primary outcome

This investigation predominantly incorporated variables such as the age, gender, BMI, marital status, ethnic groups, Glasgow Coma Scale (SOFA), acute physiology scores III (APSIII), Charlson score, and Glasgow Coma Scale (GCS) of sepsis patients. The initial vital signs measured within 24 h included systolic blood pressure (SBP), diastolic blood pressure (DBP), mean blood pressure (MBP), heart rate (HR), respiratory rate (RR), temperature (T), and oxygen saturation (SPO_2_). The repeated heart rate measurements were taken consecutively for 10 h post-ICU admission, documented hourly. In instances of multiple measurements within an hour, the initial reading was recorded. Laboratory metrics gauged within the first 24 h encompassed hematocrit, platelets, white blood cell count (WBC), bicarbonate, blood urea nitrogen (BUN), creatinine, lymphocytes, neutrophils, prothrombin time (PT), partial thromboplastin time (PTT), international normalized ratio (INR), and lactate. Existing health conditions accounted for were myocardial infarction (MI), congestive heart failure (CHF), peripheral vascular disease (PVD), chronic pulmonary disease (CPD), diabetes, and liver disease, vasoactive agent, renal replacement therapy (RRT), blood transfusion, invasive mechanical ventilation, source of infection, β-blockers. The outcome of this study was the short-term prognosis of patients with sepsis, including all-cause mortality at 30 days.

### Statistical analysis

Missing values are a common problem in public databases, and for this study, we only retained data with less than 10% missing values. For handling missing covariable values, we employed the “mice” package in R software, utilizing multiple imputation. After testing, none of the continuous variables in this study conformed to normal distribution, so they were expressed as median and interquartile range (IQR) and analyzed by Kruskal-Wallis test. Categorical variables were expressed as numbers and percentages, as well as differences between groups were determined using the chi-square test.

Latent Growth Mixture Modeling (LGMM) is extensively utilized, primarily for evaluating longitudinal data. Through the “lcmm” package in R software, we scrutinized the heart rate trajectory via the development of an LGMM. The model postulates that within a population, there exist distinct latent categories, each maintaining a uniform trajectory, typified by its mean trajectory profile [12]. Within the formulation of the LGMM, the paramount consideration is determining the optimal number of latent categories. To pinpoint the best latent categories for the LGMM, we initially set up a quadratic growth model. Building upon this foundation, we incrementally added categories, culminating in the establishment of seven distinct groups. Criteria for gauging the optimal categories of LGMM include: First, the lower the Akaike information criterion, Bayesian information Criteria (BIC), and sample-adjusted BIC are, the better the fitting effect will be [13]. Previous study had found that SABIC is the best information indicator [14]. We determined the optimal number of classes according to the principle that a lower SABIC. Secondly, the higher the log-likelihood ratio and entropy are, the better the fitting effect will be, and the entropy is greater than 0.7. Thirdly, the value of the posterior probability was between 0 and 1, and the closer it was to 1, the more accurate the classification was the sample size of each class shall greater than 1% of the total sample size, and the average posterior probability of each class is greater than 70%.

The Kaplan-Meier (K-M) method was used to draw cumulative incidence curves showing the occurrence of deaths in different groups of sepsis patients at follow-up, and the log-rank test was used to compare the differences in risk between the different groups. Cox proportional-hazards regression was constructed to identify differences in short-outcome mortality risk between patients in different trajectory categories, and the results were expressed as hazard ratios (HRs) and 95% confidence intervals (95% CIs). Model I adjusted for no adjusting for any factor. Model II adjusted for age and gender. The optimal regularization parameter λ was determined using 10-fold cross-validation of LASSO regression, and model fitting was performed using the glmnet package to screen for model III-adjusted confounders. The collinearity between variables was assessed using variance inflation factor (VIF).

A significant difference was defined as P value < 0.05 (2-tailed). All statistical analyses were performed using R software (version 4.1.0) and EmpowerStats statistical software (http://www.empowerstats.com, X&Y Solutions, Inc. Boston, MA) in this study.

## Result

### Baseline characteristics

A total of 5,511 patients were included in the study, 58.8% of whom were male. The SOFA score was 9.0 (range 6.0–12.0), with CHF being the most common comorbidity at 39.2%. Further details of general population characteristics can be found in Table 1.

### Latent growth mixture modeling

The goodness-of-fit statistics for the LGMM model are presented in Table 2. Across the models from one to six classes in LGMM, the log-likelihood ratio demonstrated an increasing trend, whereas the AIC, BIC, and SABIC consistently decreased. The six-class model had an entropy value greater than 0.9, which, although lower than that of models with one to four classes, was higher than the five-class model. We constructed six models, in which the smallest class in the sixth model accounted for approximately 2.67% of the population, surpassing the general requirement for trajectory models that the sample size should be at least 1%, thus meeting the standard. The average posterior probabilities for the one to six-class models were 94.22%, 90.74%, 95.37%, 93.31%, 95.77%, and 96.36%, respectively (Table S1). Each class had an average value exceeding 70%, underscoring the reliability of the results. Therefore, the six-class model emerges as the optimal choice.

**Fig. 1:**
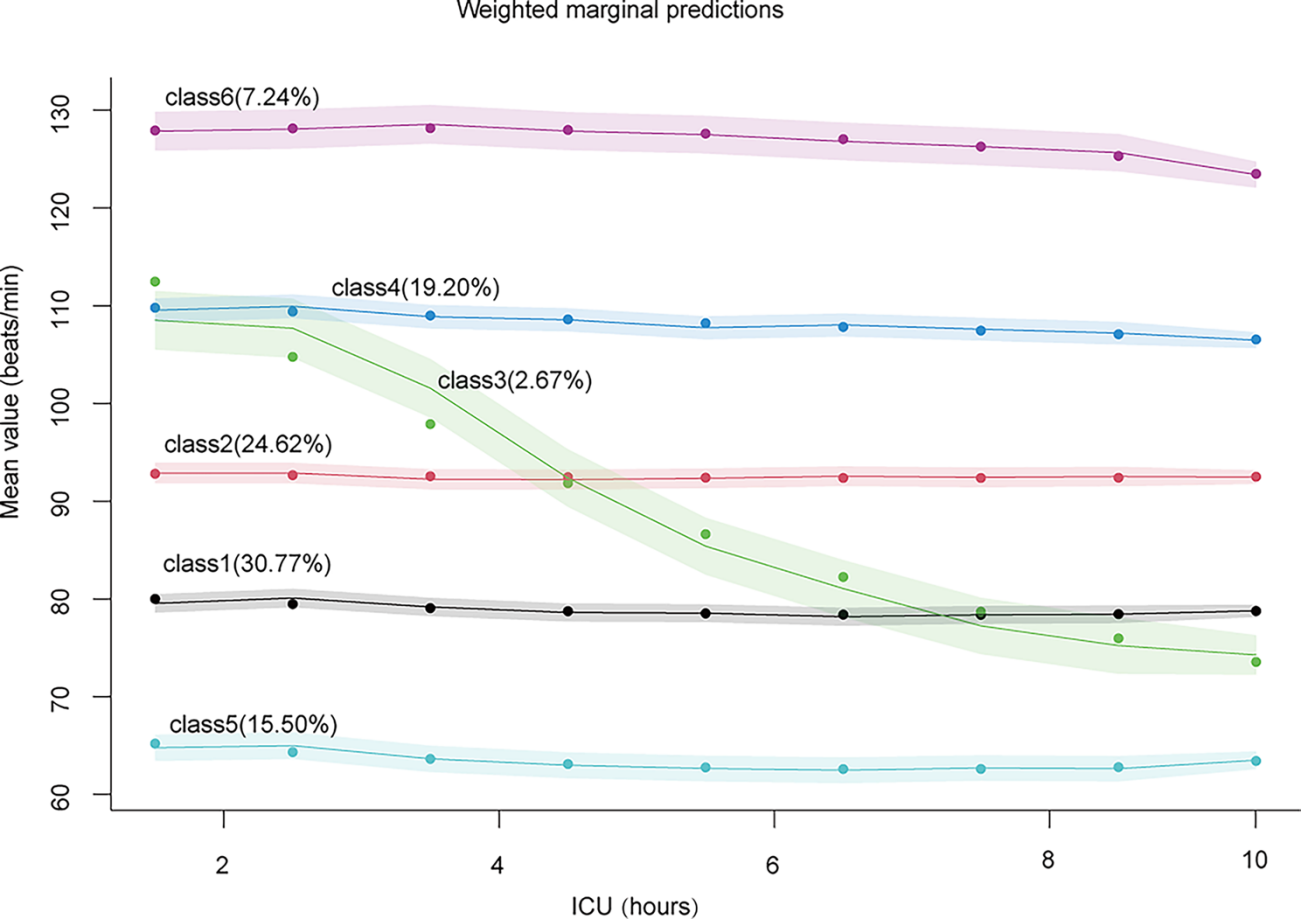
Six classes identified by trajectories of heart rate. The shaded area indicates the 95% confidence interval for each mean trajectory. The percentages in the parentheses indicate the percentages of patients each class accounts for

The heart rate trajectories for the six-class models are depicted in Fig. 1. The first class, representing the majority, accounts for 30.77% of the total sample, with a stable heart rate fluctuating around 80 beats per minute. The second class, slightly smaller, constitutes 24.62% of the sample, maintaining a consistent heart rate at about 92 beats per minute. The third class, the smallest, comprises 2.67% of the total, beginning with a brief stabilization followed by a decline, marking it as the category with the most significant fluctuation among the six. The fourth class makes up 19.20% of the total, with a steady heart rate around 110 beats per minute. The fifth class, accounting for 15.50%, has the lowest amplitude, oscillating near 65 beats per minute. The sixth class is 7.24% and has the highest heart rate at around 128 beats per minute.

They were stratified based on six trajectory classifications, with baseline characteristics presented in Table 1. The findings reveal that the average age of those in Class 5 is 69.0 years, making it the oldest group among all classifications. When examining the gender distribution within each class, males constitute approximately 58.8% of this class. BMI results indicate that patients in each category are classified as obese, with an average BMI exceeding 28.0 kg/m^2^. Additional baseline characteristics are shown in Table 1.

### Survival analysis

**Fig. 2:**
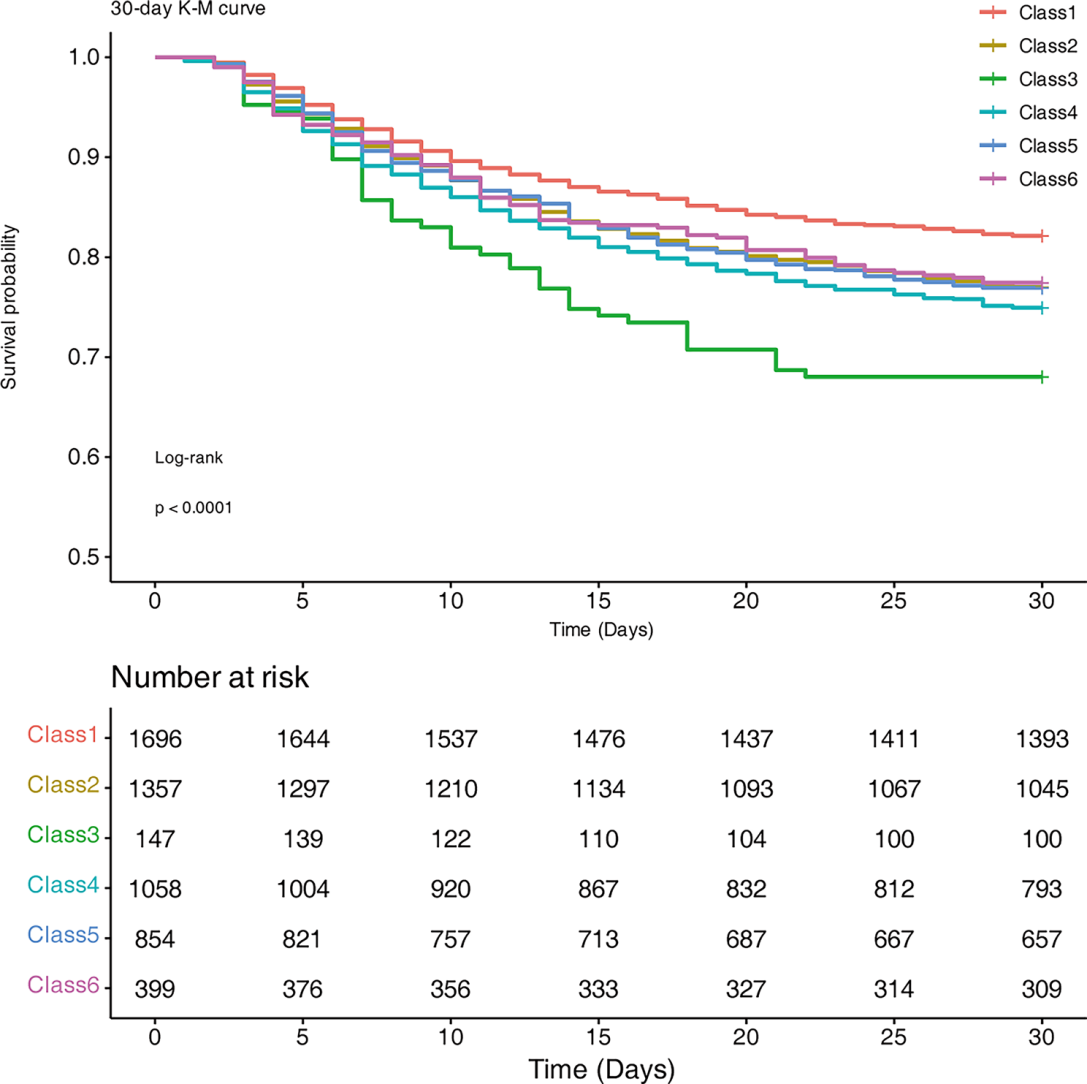
Kaplan-Meier analyzed the cumulative risk of death in patients with sepsis at 30-day

The Kaplan-Meier survival curve revealed a marked disparity in survival probabilities across the classes (Fig. 2). Notably, Class 3 exhibited the highest mortality rate, while Class 1 boasted the lowest. Furthermore, the log-rank test underscored a significant variation in survival probabilities among the classes (*P* < 0.001).

### Cox proportional-hazards regression model

**Fig. 3:**
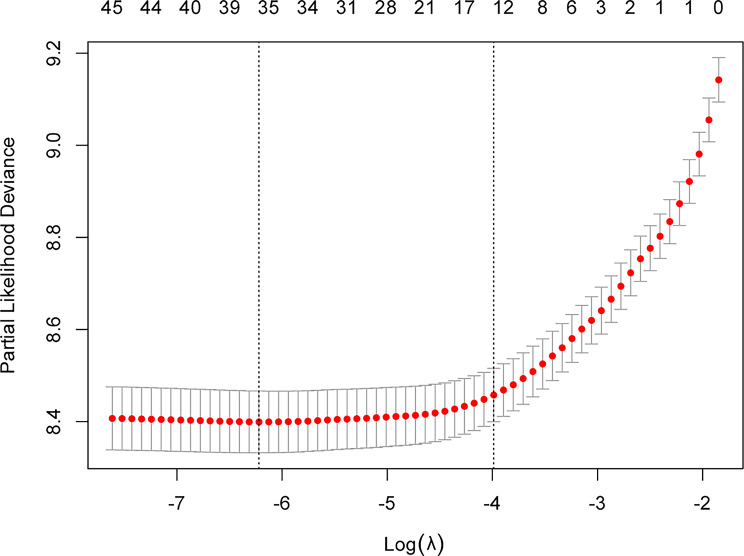
Different mean-squared error across the range of lambda. The mean-squared error was estimated with cross-validation technique and the largest lambda value was chosen when the cross-validation error was within one standard error of the minimum

To censor out the variables adjusted for Model III, an initial screening of the variables was performed using LASSO. Figure 3 shows the different mean square errors in the log(λ) range. When the cross-validation error is less than the standard error of the minimum value, the maximum λ value is chosen. Twelve variables were retained in the model: marital status, cardiovascular disease, ethnic groups, live disease, Charlson score, APSIII, age, hemoglobin, PT, neutrophils, and PTT and T (Figure S1-S2). The Vif between variables is less than 2, indicating that there is no collinearity (Table S2). The outcomes derived from the Cox proportional-hazards regression analysis are delineated in Table 3. According to adjust the Model I and using Class 1 as a reference, the results of Classes 2–6 are as follows: 1.32 (1.13, 1.55), 1.96 (1.44, 2.67), 1.47 (1.24, 1.73), 1.33 (1.11, 1.59), and 1.30 (1.02, 1.64). According to adjust the Model II, the results of Classes 2–6 as follows: 1.42 (1.22, 1.67), 2.08 (1.53, 2.82), 1.71 (1.45, 2.02), 1.28 (1.07, 1.53), and 1.69 (1.33, 2.15). According to adjusted the model III, the results of Classes 2–6 as follows: 1.28 (1.09, 1.50), 1.88 (1.38, 2.56), 1.49 (1.26, 1.76), 1.24 (1.03, 1.48), and 1.30 (1.02, 1.66). Data from Table 3 indicates that sepsis patients in Class 3, characterized by their heart rate trajectory, manifest the highest mortality rate within a 30-day outcome. Conversely, Class 1 has the lowest mortality trajectory. Statistically significant disparities are evident between these two classes, with p-values consistently below 0.05.

## Discussion

In this retrospective study, we discerned that categorizing septic patients admitted to the ICU into six heart rate classes yielded significant insights. Specifically, patients whose heart rate fluctuated around 80 beats per minute during the initial 10 h were associated with improved short-term prognosis. This study further revealed that during the first 10 h following ICU admission, most septic patients exhibited relatively stable heart rates, suggesting that despite critical illness, intrinsic heart rate regulation may demonstrate unexpected stability in early sepsis. This early hemodynamic stability could represent a critical time window during which precise monitoring or targeted interventions might significantly influence subsequent clinical outcomes. Crucially, even subtle disruptions to this stability may herald clinical deterioration. This finding contrasts with conventional perceptions of early sepsis as a state of inherent heart rate volatility, underscoring its substantial clinical and pathophysiological significance.

The heart rate is one of the most fundamental vital signs and is among the most readily accessible clinical metrics. The cardiac electrical activity originates from the sinoatrial node located in the right atrium of the heart, primarily generating four distinct currents. The parasympathetic and sympathetic nerves release acetylcholine and norepinephrine, respectively, which act on muscarinic and adrenergic receptors, to modulate heart rate. Acetylcholine binds to muscarinic receptors in the heart, resulting in a decreased heart rate, while norepinephrine binds to adrenergic receptors, leading to an increased heart rate. This is attributed to the paramount role of the vagus nerve system in heart rate regulation; it exerts an inhibitory effect on the heart, reducing the pace of heartbeats. Conversely, when norepinephrine is released, it binds to the heart’s adrenergic receptors, inducing an increased heart rate. The sympathetic nervous system typically activates during stress and excitement, augmenting both cardiac contractility and heart rate pace [15]. In the early stages of sepsis, tachycardia, or an accelerated heart rate, is commonly observed clinically. This is often perceived as the heart’s compensatory mechanism to maintain adequate cardiac output. Such tachycardia is associated with a diminished vagal tone in sepsis patients and an enhanced sensitivity to adrenergic stimulation due to lipopolysaccharides [16, 17].

In previous animal studies conducted by Joulin O et al. [18], it was discerned that lipopolysaccharides (LPS) impede the phosphorylation of phospholamban, affecting both the cardiac force-frequency relationship and the frequency-dependent acceleration of relaxation. The perturbation of left ventricular relaxation’s frequency-dependent acceleration, which conventionally aids in optimal heart cavity filling, might be deleterious in sepsis—a condition often characterized by heightened heart rates and a reliance on preload. In a 28-day study involving patients with MODS induced by either sepsis or non-sepsis, Hoke and colleagues discerned that sepsis patients with a heart rate of less than 90 beats per minute fared notably better than those exceeding 90 beats per minute [19]. Furthermore, the prognosis markedly improved upon the administration of heart rate reduction therapeutics [20], and prior meta-analyses investigating the impact of ultra-short-acting β-blockers on mortality in sepsis patients with persistent tachycardia have revealed that administration of these agents (such as esmolol and landiolol) significantly reduces 28-day mortality in septic patients exhibiting persistent tachycardia following initial resuscitation [21]. However, according to the 2025 German Sepsis Guidelines, the routine use of β-blockers is not recommended [22]. This position is further supported by recent studies reporting inconsistent findings regarding their mortality benefits [23, 24].As a result, the role of β-blockers in sepsis remains a subject of ongoing debate. Nevertheless, potential advantages associated with ultrashort-acting β-blockers may be attributed to the following mechanisms. This phenomenon may be attributable to the ability of β-blockers to suppress pro-inflammatory cytokines, mitigate the systemic inflammatory response [25], and improve metabolic profiles among sepsis patients [26].Our findings indicate that the cohort receiving β-blocker intervention (Class 1) was larger than other classes and demonstrated a lower 30-day mortality risk. However, the therapeutic efficacy of β-blockers in reducing heart rate among sepsis patients remains controversial. Notably, research has shown that esmolol fails to improve 28-day and 90-day mortality rates in sepsis [27]. Furthermore, it may reduce diastolic and mean arterial pressures while increasing vasopressor requirements [28]. This lack of mortality benefit with esmolol is corroborated by the meta-analysis conducted by Sato R et al. [29]. In the realm of cardiac diseases, utilizing ivabradine to treat systolic heart failure and reduce heart rate has been evidenced to significantly diminish both cardiovascular mortality and rehospitalization rates due to heart failure [30]. In another study, Ivabradine administered to MODS patients in a randomized trial reduced heart rate modestly by 7 beats/min., but had no effect on haemodynamics or disease severity [31]. Our study indicates that when the heart rate of septic patients remains within the ‘class 6’ category at 128 beats per minute, it poses a heightened risk and is deemed a perilous factor with an HR (CI %) of 1.30(1.02, 1.66). Notably, within our study cohort, the class 3 group exhibited significant heart rate volatility, with a precipitous decline in the short term. The findings suggest that this category of septic patients faces the gravest risks, with an HR (CI%) of 1.88(1.38, 2.56). We believe that there may be several pathophysiological mechanisms. One is that infectious cardiomyopathy leads to ventricular dilation and reduced ejection fraction, and then develops into bradycardia at the end of cardiogenic shock, indicating circulatory failure and poor prognosis [32]. Another possible explanation is autonomic nerve dysfunction caused by sepsis. The inflammatory reaction destroys the balance between the sympathetic and parasympathetic nerves, impairing the regulation of heart rate. As the disease progresses, the loss of autonomic control prevents compensatory tachycardia from being maintained, leading to delayed bradycardia [33]. Additionally, the action of drugs may also be effective. Common ICU medications such as sedatives and beta-blockers can suppress sinoatrial node activity [34]. Therefore, the above pathophysiological mechanisms may reduce the heart rate of patients with class 3, and are associated with a poor prognosis. Intriguingly, our institution identified that when heart rate trajectories hover around 80 beats per minute, this category of septic patients exhibits the lowest risk, the most favorable prognosis, and the highest short-term survival rate. Our findings align closely with the research conducted by Beesley SJ et al. [20]. However, their study incorporated only an hour of patient heart rate data, whereas ours examined ten consecutive hours of heart rate in septic patients, thereby establishing a short-term developmental trajectory that offers more significant clinical insights. Another study in the study of people with acute myocardial infarction found that when the heart rate was greater than 80 beats/hour, it was an independent risk factor for death in hospital [35].

### Strengths and limitations of the study

This study explores firstly the relationship between heart rate trajectories and the short-term prognosis of sepsis patients. The extensive data sample furnishes dependable outcomes for our research. Nevertheless, our investigation bears certain limitations. Firstly, despite our efforts to control for potential confounding factors, residual confounding remains an issue. Specifically, heart rate trajectories may be influenced by underlying disease severity and treatment-related factors such as fluid resuscitation, vasopressors, sedatives, and beta-blockers. Therefore, we cannot determine whether heart rate trajectories themselves represent a causal or merely a marker of disease severity and treatment response. We hope future studies will allow for a deeper exploration of these mechanisms. Secondly, MIMIC-IV is a single-center database, and this study has selection bias which limits the extrapolation of our conclusions. Thirdly, given the retrospective observational design of this study, our findings only describe correlations, which could be analyzed in the future with mediation analysis or causal studies. Fourthly, we did not specifically divide the patients into sepsis and septic shock subgroups for the study. Fifthly, this study evaluated heart rate trajectories within 10 h of ICU admission. This timeframe was chosen to capture the early recovery phase and minimise the impact of later treatment interventions. However, alternative timeframes, such as six hours or 24 h, or the post-resuscitation period, may provide different prognostic insights. The validity of this timeframe remains to be confirmed and can be verified through future external or prospective studies. Sixthly, we did not conduct further studies on the effects of vasoactive drugs and beta-blockers on outcomes. Finally, we only discussed the influence of the trajectory in the changes in heart rate of a single indicator on the occurrence of sepsis patients and in future studies, the combined trends of other relevant indicators can be utilized to predict the onset of diseases.

## Conclusion

In conclusion, maintaining a sustained heart rate of approximately 80 beats per minute during the initial 10-hour period post-ICU admission may be associated with improved short-term outcomes in patients with sepsis.

**Table 1:** Baseline characteristics of six classes

Variables	overall	class1	class2	class3	class4	class5	class6	*P*-value
N	5511	1696	1357	147	1058	854	399	
Age (year)	65.0 (54.0–76.0)	68.0 (58.0–77.0)	65.0 (53.0–75.0)	66.0 (54.5–77.5)	60.0 (47.0–72.0)	69.0 (60.0–78.0)	55.0 (41.0–68.0)	< 0.001
Male, n (%)	3239 (58.8)	1016 (59.9)	812 (59.8)	84 (57.1)	588 (55.6)	506 (59.3)	233 (58.4)	< 0.001
BMI (kg/m^2^)	28.4 (24.5–33.9)	28.4 (24.5–33.8)	28.3 (24.6–33.8)	27.4 (23.1–33.9)	28.3 (24.4–33.8)	28.5 (24.6–34.0)	28.5 (24.7–34.6)	0.54
**Marital status**,** n (%)**								< 0.001
married	2281 (41.4)	784 (46.2)	549 (40.5)	48 (32.7)	393 (37.1)	371 (43.4)	136 (34.1)	
single	1594 (28.9)	408 (24.1)	412 (30.4)	51 (34.7)	343 (32.4)	215 (25.2)	165 (41.4)	
other	1636 (29.7)	504 (29.7)	396 (29.2)	48 (32.7)	322 (30.4)	268 (31.4)	98 (24.6)	
**Ethnic groups**,** n (%)**								< 0.001
white	3519 (63.9)	1111 (65.5)	878 (64.7)	86 (58.5)	650 (61.4)	554 (64.9)	240 (60.2)	
black	525 (9.5)	145 (8.5)	131 (9.7)	16 (10.9)	113 (10.7)	81 (9.5)	39 (9.8)	
other	1467 (26.6)	440 (25.9)	348 (25.6)	45 (30.6)	295 (27.9)	219 (25.6)	120 (30.1)	
**Severity scales**								
SOFA	9.0 (6.0–12.0)	8.0 (6.0–11.0)	9.0 (6.0–12.0)	9.0 (7.0–12.0)	9.0 (6.0–12.0)	8.0 (6.0–11.0)	10.0 (6.0, 13.0)	< 0.001
APSIII	64.0 (46.0–85.0)	56.0 (40.0–78.0)	66.0 (49.0–88.0)	69.0 (53.5–85.5)	70.0 (53.2–92.0)	58.5 (44.0–78.0)	78.0 (60.0-103.0)	< 0.001
Charlson score	5.0 (4.0–7.0)	6.0 (4.0–8.0)	6.0 (4.0–7.0)	5.0 (3.0–7.0)	5.0 (3.0–7.0)	6.0 (4.0–8.0)	4.0 (2.0–6.0)	< 0.001
GCS	11.0 (6.0–14.0)	12.0 (7.0–14.0)	10.0 (6.0–14.0)	10.0 (6.0–14.0)	11.0 (7.0–14.0)	11.0 (6.0–14.0)	11.0 (6.0–14.0)	< 0.001
**Vital sign**								
SBP (mmHg)	111.0 (103.0-120.0)	111.0 (104.0-120.0)	110.0 (103.0-119.0)	110.0 (104.0-119.5)	109.0 (101.0-118.0)	115.0 (107.0-126.0)	107.0 (100.0-115.0)	< 0.001
DBP (mmHg)	60.0 (54.0–66.0)	58.0 (52.0–64.0)	60.0 (55.0–67.0)	61.0 (56.0-67.5)	62.0 (57.0–68.0)	58.0 (52.0–64.0)	63.0 (58.0-69.5)	< 0.001
MBP (mmHg)	75.0 (70.0–81.0)	74.0 (69.0–80.0)	75.0 (70.0–81.0)	75.0 (70.5–82.0)	75.0 (70.0–82.0)	75.0 (69.0–81.0)	76.0 (71.0–82.0)	< 0.001
HR (beats/min)	87.0 (75.0-10.02)	79.0 (73.0–84.0)	92.0 (87.0–98.0)	85.0 (74.0-100.0)	108 0.0 (102.0-114.0)	63.0 (58.0–69.0)	125.0 (119.0-134.0)	< 0.001
RR (times/min)	20.0 (17.0–23.0)	19.0 (17.0–21.0)	20.0 (18.0–23.0)	20.0 (17.0, 22.5)	22.0 (19.0–25.0)	18.0 (16.0–21.0)	23.0 (19.0-26.5)	< 0.001
T (℃)	36.9 (36.6–37.4)	36.8 (36.5–37.2)	36.9 (36.6–37.4)	36.9 (36.5–37.3)	37.1 (36.7–37.6)	36.8 (36.4–37.1)	37.3 (36.8–37.7)	< 0.001
SPO_2_ (%)	98.0 (96.0–99.0)	98.0 (97.0–99.0)	98.0 (96.0–99.0)	98.0 (96.0–99.0)	97.0 (95.0–99.0)	98.0 (97.0–99.0)	97.0 (95.0–98.0)	< 0.001
**Laboratory tests**								
Hematocrit (%)	34.9 (30.7–39.9)	34.4 (30.7–38.7)	34.6 (30.5–39.7)	34.2 (30.1–40.8)	35.3 (30.7–40.4)	35.4 (30.5–40.3)	36.6 (32.0-40.9)	< 0.001
Platelets (×10^9/L)	155.0 (104.5, 222.0)	147.0 (105.0-212.2)	155.0 (102.0-220.0)	166.0 (102.0-247.0)	164.0 (105.0-238.0)	158.0 (110.0-216.0)	153.0 (90.0-235.0)	0.017
WBC (×10^9/L)	15.6 (11.2–20.8)	15.2 (11.3–20.1)	16.1 (11.7–21.4)	16.2 (10.2–22.9)	16.3 (11.2–22.0)	13.8 (10.3–18.3)	17.2 (12.3–23.6)	< 0.001
Bicarbonate (mEq/L)	24.0 (21.0–27.0)	24.0 (22.0–27.0)	24.0 (21.0–27.0)	23.0 (20.0, 27.0)	23.0 (20.2–26.0)	24.0 (21.0–27.0)	23.0 (20.0–25.0)	< 0.001
BUN (mg/dL)	26.0 (17.0–44.0)	25.0 (17.0–41.0)	28.0 (17.0–47.0)	28.0 (19.0–41.0)	27.0 (17.0–45.0)	27.0 (18.0–45.0)	28.0 (16.0–46.0)	0.009
Creatinine (g/dL)	1.3 (0.9–2.3)	1.3 (0.9–2.1)	1.4 (0.9–2.4)	1.2 (0.8-2.0)	1.4 (0.9–2.4)	1.3 (0.9–2.1)	1.5 (1.0-2.5)	< 0.001
Lymphocytes (×10^9/L)	1.2 (0.7–1.8)	1.3 (0.8–1.9)	1.1 (0.7–1.8)	1.1 (0.7–1.9)	1.1 (0.7–1.7)	1.1 (0.7–1.8)	1.0 (0.6–1.6)	< 0.001
Monocytes (×10^9/L)	0.6 (0.4-1.0)	0.6 (0.3–0.9)	0.6 (0.4-1.0)	0.7 (0.4–1.1)	0.6 (0.4–1.1)	0.6 (0.4–0.9)	0.8 (0.4–1.2)	0.121
Neutrophils (×10^9/L)	11.0 (7.3–15.8)	10.4 (7.0-14.8)	11.4 (7.8–16.4)	12.3 (7.2–17.0)	11.6 (7.5–16.9)	9.8 (6.6–14.1)	12.8 (8.1–18.4)	0.201
INR	1.4 (1.2–1.8)	1.4 (1.2–1.8)	1.4 (1.2–1.9)	1.4 (1.2–1.8)	1.4 (1.2–1.9)	1.3 (1.2–1.7)	1.5 (1.3–2.1)	< 0.001
PT (s)	15.6 (13.4–19.9)	15.5 (13.4–19.1)	15.7 (13.6–20.2)	15.5 (13.0-19.6)	15.7 (13.5–20.9)	14.8 (12.9–18.7)	16.9 (14.4–23.1)	< 0.001
PTT (s)	35.5 (29.5–51.0)	35.1 (29.5–48.2)	35.1 (29.3–51.2)	33.8 (28.6–50.3)	35.8 (29.6–51.0)	35.4 (29.5–51.4)	40.0 (31.6–62.2)	0.100
Lactate (mmol/L)	2.5 (1.6–4.3)	2.4 (1.6–3.8)	2.5 (1.6–4.8)	2.6 (1.7–4.3)	2.6 (1.6–4.6)	2.1 (1.4–3.4)	3.6 (2.1–5.8)	< 0.001
**Comorbidities**,** n (%)**								
MI	1229 (22.3)	400 (23.6)	319 (23.5)	31 (21.1)	207 (19.6)	215 (25.2)	57 (14.3)	< 0.001
CHF	2160 (39.2)	723 (42.6)	525 (38.7)	52 (35.4)	385 (36.4)	338 (39.6)	137 (34.3)	< 0.001
CVD	903 (16.4)	307 (18.1)	225 (16.6)	19 (12.9)	143 (13.5)	164 (19.2)	45 (11.3)	< 0.001
CPD	1673 (30.4)	524 (30.9)	416 (30.7)	53 (36.1)	317 (30.0)	258 (30.2)	105 (26.3)	< 0.001
Diabetes	1879 (34.1)	597 (35.2)	481 (35.4)	46 (31.3)	336 (31.8)	295 (34.5)	124 (31.1)	< 0.001
Live disease	1025 (18.6)	221 (13.0)	269 (19.8)	31 (21.1)	271 (25.6)	114 (13.3)	119 (29.8)	< 0.001
**Treatment**								
Ventilation_status, n (%)								< 0.001
invasive vent	1701 (30.9)	315 (18.6)	445 (32.8)	25 (17.0)	487 (46.3)	342 (40.0)	87 (21.8)	
supplemental oxygen	642 (11.6)	104 (8.3)	125 (9.2)	12 (8.2)	163 (15.4)	198 (23.2)	40 (10.0)	
tracheostomy	1901 (34.5)	1062(60.5)	489 (35.99)	52 (35.34)	38 (3.60)	59 (6.81)	201(50.2)	
other	1701 (30.9)	315 (18.5)	445 (32.8)	25 (17.0)	487 (46.3)	342 (40.0)	87 (21.8)	
Transfusion, n (%)	631 (11.4)	84 (5)	84 (6.2)	36 (25)	238 (22.5)	164 (19.2)	25 (6.3)	< 0.001
RRT, n (%)	266 (4.8)	59 (3.5)	65 (4.8)	10 (6.8)	63 (6.0)	52 (6.1)	17 (4,3)	0.011
Vasoactive_agent, n (%)								< 0.001
norepinephrine	926 (16.8)	279 (16.5)	256 (18.9)	25 (17.0)	172 (16.3)	141 (16.5)	53 (13.3)	
epinephrine	499 (9.0)	117 (6.9)	135 (9.9)	15 (10.2)	91 (8.6)	100 (11.7)	41 (10.3)	
dopamine	312 (5.7)	76 (4.5)	90 (6.6)	9 (6.1)	60 (5.7)	47 (5.5)	30 (7.5)	
dobutamine	119 (2.2)	36 (2.1)	21 (1.5)	5 (3.4)	15 (1.4)	27 (3.2)	15 (3.8)	
Infection_source, n (%)								< 0.001
respiratory	1321 (24.0)	380(22.4)	345(25.4)	42(28.4)	272(25.7)	195(22.8)	88(22.0)	
blood	875 (15.9)	263(15.5)	228(16.8)	21(14.3)	219(20.7)	183(21.4)	61(15.3)	
stool	495 (9.0)	114(6.7)	90(6.6)	10(7.2)	87(8.2)	64(7.5)	30(7.5)	
urine	615 (11.2)	167(9.84)	162(11.9)	11(7.8)	141(13.3)	93(10.9)	41(10.3)	
other	2205 (40.0)	771(45.5)	403(29.8)	62(42.0)	338(32.0)	319(37.4)	179(44.9)	
β- blockers, n (%)								< 0.001
Esmolol	486 (8.8)	141 (8.3)	121 (8.9)	11 (7.5)	88 (8.3)	85 (10.0)	40 (10.0)	
Metoprolol	318 (5.8)	103 (6.1)	96 (7.1)	4 (2.7)	40 (3.8)	54 (6.3)	21 (5.3)	
Atenolol	158 (2.9)	70 (4.1)	43 (3.2)	2 (1.4)	33 (3.1)	13 (1.5)	10 (2.5)	
Propranolol	86 (1.6)	41 (2.4)	15 (1.1)	1 (0.7)	16 (1.5)	6 (0.7)	7 (1.8)	
others	144 (2.6)	60 (3.5)	36 (2.7)	1 (0.7)	23 (2.2)	13 (1.5)	11 (2.8)	
30-day mortality, n (%)	1214 (22.0)	303 (17.87)	312 (29.86)	47 (32.00)	265 (25.05)	197 (23.07)	90 (22.56)	< 0.001

Abbreviations: BMI body Mass Index, SOFA Sequential Organ Failure Assessment, GCS glasgow coma scale, APSIII acute rhysiological scores III, SBP systolic blood pressure, DBP diastolic blood pressure, MBP mean blood pressure, HR heart rate, RR respiratory rate, T temperature, SPO_2_ oxygen saturation of blood, WBC white blood cell, BUN blood urea nitrogen, INR international normalized ratio, PT prothrombin time, PTT partial thromboplastin time, MI myocardial infarction, CHF congestive heart failure, CVD peripheral vascular disease, CPD chronic pulmonary disease, RRT renal replacement therapy

**Table 2:** Statistics for choosing the best number of classes

Numbel of class	Log likelihood	AIC	BIC	SABIC	Entropy	%class1	%class2	%class3	%class4	%class5	%class6
1	-243687.7	487383.3	487409.8	487397.1	1.0000000	100.000000					
2	-225763.0	451542.1	451595.0	451569.6	0.9471034	37.688260	62.311740				
3	-218445.0	436914.1	436993.5	436955.3	0.9337962	30.883687	24.042823	45.07349			
4	-214381.8	428795.6	428901.4	428850.5	0.9353187	27.363455	20.141535	11.57685	40.91816		
5	-212285.8	424611.7	424743.9	424680.4	0.9210581	7.131192	25.730357	19.12539	32.19017	15.82290	
6	-211676.5	423401.0	423559.8	423483.5	0.9261932	30.774814	24.62348	2.667392	19.19797	15.49628	7.240065

AIC Akaike information criterion, BIC Bayesian information criteria, SABIC sample-adjusted information criteria

**Table 3:** Results of Cox proportional hazard models

Characteristic	Model 1			Model 2			Model 3		
	HR	95% CI	*P*	HR	95% CI	*P*	HR	95% CI	*P*
**class**									
1	Reference	Reference		Reference	Reference		Reference	Reference	
2	1.32	1.13, 1.55	< 0.001	1.42	1.22, 1.67	< 0.001	1.28	1.09, 1.50	0.002
3	1.96	1.44, 2.67	< 0.001	2.08	1.53, 2.82	< 0.001	1.88	1.38, 2.56	< 0.001
4	1.47	1.24, 1.73	< 0.001	1.71	1.45, 2.02	< 0.001	1.49	1.26, 1.76	< 0.001
5	1.33	1.11, 1.59	0.002	1.28	1.07, 1.53	0.007	1.24	1.03, 1.48	0.020
6	1.30	1.02, 1.64	0.030	1.69	1.33, 2.15	< 0.001	1.30	1.02, 1.66	0.034
P for trend			< 0.001			< 0.001			0.002

Abbreviations: CVD peripheral vascular disease, APSIII acute rhysiological scores III, T temperature, PT prothrombin time, PTT partial thromboplastin time. CI = Confidence Interval, HR = Hazard Ratio

Model 1: no covariates were adjusted

Model 2: adjusted for Age and Gender

Model 3: adjusted for Marital status, Race, CVD, Live disease, Age, Charlson score, APSIII, T, Hemoglobin, Neutrophils, PT, PTT

## Supplementary Information

Below is the link to the electronic supplementary material.

Supplementary Material 1

## Data Availability

Publicly available datasets were analysed in this study. These data can be found here. The data were available on the MIMIC-IV website at https://mimic.mit.edu/.

## Abbreviations

SMD: Standardized Mean Difference
LGMM: Latent growth mixture modeling
MI: myocardial infarction
CHF: congestive heart failure
CVD: peripheral vascular disease
CPD: chronic pulmonary disease
BMI: body Mass Index
SOFA: Sequential Organ Failure Assessment
GCS: glasgow coma scale
APSIII: acute physiology scores III
WBC: white blood cell
BUN: blood urea nitrogen
PT: prothrombin time
PTT: partial thromboplastin time
INR: international normalized ratio
SBP: systolic blood pressure
DBP: diastolic blood pressure
MBP: mean blood pressure
HR: heart rate
RR: respiratory rate
T: temperature
SPO_2_: oxygen saturation of blood
IV: MIMIC Medical Information Mart for Intensive Care IV
ICU: Intensive care unit
HR: Hazard ratio

## References

[1] Liu V, Escobar GJ, Greene JD, Soule J, Whippy A, Angus DC, Iwashyna TJ. Hospital deaths in patients with sepsis from 2 independent cohorts. JAMA. 2014;312(1):90–2. 24838355 10.1001/jama.2014.5804

[2] Adhikari NK, Fowler RA, Bhagwanjee S, Rubenfeld GD. Critical care and the global burden of critical illness in adults. Lancet. 2010;376(9749):1339–46. 20934212 10.1016/S0140-6736(10)60446-1 PMC7136988

[3] Dendoncker K, Libert C. Glucocorticoid resistance as a major drive in sepsis pathology. Cytokine Growth Factor Rev. 2017;35:85–96. 28479044 10.1016/j.cytogfr.2017.04.002

[4] Tiru B, DiNino EK, Orenstein A, Mailloux PT, Pesaturo A, Gupta A, McGee WT. The economic and humanistic burden of severe sepsis. PharmacoEconomics. 2015;33(9):925–37. 25935211 10.1007/s40273-015-0282-y

[5] Rudd KE, Johnson SC, Agesa KM, Shackelford KA, Tsoi D, Kievlan DR, Colombara DV, Ikuta KS, Kissoon N, Finfer S, et al. Global, regional, and National sepsis incidence and mortality, 1990–2017: analysis for the global burden of disease study. Lancet. 2020;395(10219):200–11. 31954465 10.1016/S0140-6736(19)32989-7 PMC6970225

[6] Reinhart K, Daniels R, Kissoon N, Machado FR, Schachter RD, Finfer S. Recognizing sepsis as a global health Priority - A WHO resolution. N Engl J Med. 2017;377(5):414–7. 28658587 10.1056/NEJMp1707170

[7] Ebelt H, Geißler I, Ruccius S, Otto V, Hoffmann S, Korth H, Klöckner U, Zhang Y, Li Y, Grossmann C, et al. Direct inhibition, but indirect sensitization of pacemaker activity to sympathetic tone by the interaction of endotoxin with HCN-channels. Clin Exp Pharmacol Physiol. 2015;42(8):874–80. 25933122 10.1111/1440-1681.12415

[8] Rivers E, Nguyen B, Havstad S, Ressler J, Muzzin A, Knoblich B, Peterson E, Tomlanovich M. Early goal-directed therapy in the treatment of severe sepsis and septic shock. N Engl J Med. 2001;345(19):1368–77. 11794169 10.1056/NEJMoa010307

[9] Johnson A, Bulgarelli L, Pollard T, Horng S, Celi LA, Mark R. MIMIC-IV (version 2.0). PhysioNet. 2022. RRID:SCR_007345. Available from: 10.13026/7vcr-e114

[10] Yang J, Li Y, Liu Q, Li L, Feng A, Wang T, Zheng S, Xu A, Lyu J. Brief introduction of medical database and data mining technology in big data era. J Evid Based Med. 2020;13(1):57–69. 32086994 10.1111/jebm.12373 PMC7065247

[11] Singer M, Deutschman CS, Seymour CW, Shankar-Hari M, Annane D, Bauer M, Bellomo R, Bernard GR, Chiche JD, Coopersmith CM, et al. The third international consensus definitions for sepsis and septic shock (Sepsis-3). JAMA. 2016;315(8):801–10. 26903338 10.1001/jama.2016.0287 PMC4968574

[12] Zhang Z, Ho KM, Gu H, Hong Y, Yu Y. Defining persistent critical illness based on growth trajectories in patients with sepsis. Crit Care. 2020;24(1):57. 32070393 10.1186/s13054-020-2768-z PMC7029548

[13] Eriksson J, Nelson D, Holst A, Hellgren E, Friman O, Oldner A. Temporal patterns of organ dysfunction after severe trauma. Crit Care. 2021;25(1):165. 33952314 10.1186/s13054-021-03586-6 PMC8101241

[14] Kim SY. Determining the number of latent classes in Single- and Multi-Phase growth mixture models. Struct Equ Model. 2014;21(2):263–79. 10.1080/10705511.2014.882690 PMC3979564 24729675

[15] Levy MN. Autonomic interactions in cardiac control. Ann N Y Acad Sci. 1990;601:209–21. 2221687 10.1111/j.1749-6632.1990.tb37302.x

[16] Zorn-Pauly K, Pelzmann B, Lang P, Mächler H, Schmidt H, Ebelt H, Werdan K, Koidl B. Müller-Werdan U: endotoxin impairs the human pacemaker current if. Shock. 2007;28(6):655–61. 18092381

[17] Schmidt H, Müller-Werdan U, Hoffmann T, Francis DP, Piepoli MF, Rauchhaus M, Prondzinsky R, Loppnow H, Buerke M, Hoyer D, et al. Autonomic dysfunction predicts mortality in patients with multiple organ dysfunction syndrome of different age groups. Crit Care Med. 2005;33(9):1994–2002. 16148471 10.1097/01.ccm.0000178181.91250.99

[18] Joulin O, Marechaux S, Hassoun S, Montaigne D, Lancel S, Neviere R. Cardiac force-frequency relationship and frequency-dependent acceleration of relaxation are impaired in LPS-treated rats. Crit Care. 2009;13(1):R14. 19196490 10.1186/cc7712 PMC2688131

[19] Hoke RS, Müller-Werdan U, Lautenschläger C, Werdan K, Ebelt H. Heart rate as an independent risk factor in patients with multiple organ dysfunction: a prospective, observational study. Clin Res Cardiol. 2012;101(2):139–47. 22048696 10.1007/s00392-011-0375-3

[20] Beesley SJ, Wilson EL, Lanspa MJ, Grissom CK, Shahul S, Talmor D, Brown SM. Relative bradycardia in patients with septic shock requiring vasopressor therapy. Crit Care Med. 2017;45(2):225–33. 27618277 10.1097/CCM.0000000000002065 PMC5512273

[21] Hasegawa D, Sato R, Prasitlumkum N, Nishida K, Takahashi K, Yatabe T, Nishida O. Effect of Ultrashort-Acting β-Blockers on mortality in patients with sepsis with persistent tachycardia despite initial resuscitation: A systematic review and Meta-analysis of randomized controlled trials. Chest. 2021;159(6):2289–300. 33434497 10.1016/j.chest.2021.01.009

[22] Brunkhorst FM, Adamzik M, Axer H, Bauer M, Bode C, Bone HG, Brenner T, Bucher M, David S, Dietrich M et al. [S3 guideline on sepsis-prevention, diagnosis, therapy, and follow-up care-update 2025]. Med Klin Intensivmed Notfmed 2025. 10.1007/s00063-025-01317-1 PMC12708846 40824313

[23] Rehberg S, Frank S, Černý V, Cihlář R, Borgstedt R, Biancofiore G, Guarracino F, Schober A, Trimmel H, Pernerstorfer T, et al. Landiolol for heart rate control in patients with septic shock and persistent tachycardia. A multicenter randomized clinical trial (Landi-SEP). Intensive Care Med. 2024;50(10):1622–34. 39297945 10.1007/s00134-024-07587-1 PMC11447033

[24] Alexandru MG, Niewald P, Krüger S, Borgstedt R, Whitehouse T, Singer M, Rehberg S, Scholz SS. Mortality in septic patients treated with short-acting betablockers: a comprehensive meta-analysis of randomized controlled trials. Crit Care. 2024;28(1):392. 39605034 10.1186/s13054-024-05174-w PMC11603935

[25] Hong SY, Lai CC, Teng NC, Chen CH, Hsu CC, Chan NJ, Wang CY, Wang YH, Lin YS, Chen L. Premorbid use of selective beta-blockers improves sepsis incidence and course: human cohort and animal model studies. Front Med (Lausanne). 2023;10:1105894. 37144032 10.3389/fmed.2023.1105894 PMC10151496

[26] Schneider L, Chalmers D, O’Beirn S, Greenberg M, Cave G. Premorbid beta Blockade in sepsis is associated with a lower risk of a lactate concentration above the lactate threshold, a retrospective cohort study. Sci Rep. 2022;12(1):20843. 36460714 10.1038/s41598-022-25253-8 PMC9718750

[27] Ge CL, Zhang LN, Ai YH, Chen W, Ye ZW, Zou Y, Peng QY. Effect of β-blockers on mortality in patients with sepsis: A propensity-score matched analysis. Front Cell Infect Microbiol. 2023;13:1121444. 37056709 10.3389/fcimb.2023.1121444 PMC10086225

[28] Liang Q, Li L, Chen K, An S, Deng Z, Li J, Zhou S, Chen Z, Zeng Z, An S. Effect of Esmolol on clinical outcomes in critically ill patients: data from the MIMIC-IV database. J Cardiovasc Pharmacol Ther. 2023;28:10742484231185985. 37415421 10.1177/10742484231185985

[29] Sato R, Messina S, Hasegawa D, Santonocito C, Scimonello G, Sanfilippo G, Morelli A, Dugar S, Sanfilippo F. Mortality in patients with sepsis treated with Esmolol or landiolol: A systematic review and Meta-Analysis of randomized controlled trials with trial sequential analysis. Chest. 2025;167(1):121–38. 39197514 10.1016/j.chest.2024.08.020

[30] Swedberg K, Komajda M, Böhm M, Borer JS, Ford I, Dubost-Brama A, Lerebours G, Tavazzi L. Ivabradine and outcomes in chronic heart failure (SHIFT): a randomised placebo-controlled study. Lancet. 2010;376(9744):875–85. 20801495 10.1016/S0140-6736(10)61259-7

[31] Nuding S, Schröder J, Presek P, Wienke A, Müller-Werdan U, Ebelt H, Werdan K. Reducing elevated heart rates in patients with multiple organ dysfunction syndrome with the if (Funny channel Current) inhibitor ivabradine. Shock. 2018;49(4):402–11. 28930912 10.1097/SHK.0000000000000992

[32] Stevens DL, Bryant AE. Complexities of cardiomyopathy in septic shock. Curr Opin Infect Dis. 2025;38(3):214–21. 40127058 10.1097/QCO.0000000000001102

[33] Carrara M, Ferrario M, Bollen Pinto B, Herpain A. The autonomic nervous system in septic shock and its role as a future therapeutic target: a narrative review. Ann Intensive Care. 2021;11(1):80. 33999297 10.1186/s13613-021-00869-7 PMC8128952

[34] Guarracino F, Cortegiani A, Antonelli M, Behr A, Biancofiore G, Del Gaudio A, Forfori F, Galdieri N, Grasselli G, Paternoster G, et al. The role of beta-blocker drugs in critically ill patients: a SIAARTI expert consensus statement. J Anesth Analg Crit Care. 2023;3(1):41. 37872608 10.1186/s44158-023-00126-2 PMC10591347

[35] Davidovic G, Iric-Cupic V, Milanov S, Dimitijevic A, Petrovic-Janicijevic M. When heart goes BOOM to fast. Heart rate greater than 80 as mortality predictor in acute myocardial infarction. Am J Cardiovasc Dis. 2013;3(3):120–8. 23991346 PMC3751677

