# Supplementary material for: Influence of heart rate trajectory in 30-day mortality in sepsis patients: a retrospective study based on the MIMIC-IV database
Source: BMC Infect Dis. 2025 Dec 9;25:1702. doi: 10.1186/s12879-025-11961-9 (PMC12690948; doi:10.1186/s12879-025-11961-9)
Supplement: Supplementary file 1 — Supplementary Material 1 [file 12879_2025_11961_MOESM1_ESM.docx]

**Table S1** Mean of posterior probabilities in each class

| class | Probability1 | Probability2 | Probability3 | Probability4 | Probability5 | Probability6 |
| --- | --- | --- | --- | --- | --- | --- |
| class1 | 0.9422 | 0.0050 | 0.0000 | 0.0332 | 0.0193 | 0.0000 |
| class2 | 0.0454 | 0.9074 | 0.0060 | 0.0410 | 0.0001 | 0.0000 |
| class3 | 0.0000 | 0.0003 | 0.9537 | 0.0345 | 0.0000 | 0.0115 |
| class4 | 0.0386 | 0.0075 | 0.0208 | 0.9331 | 0.0000 | 0.0000 |
| class5 | 0.0423 | 0.0000 | 0.0000 | 0.0000 | 0.9577 | 0.0000 |
| class6 | 0.0000 | 0.0000 | 0.0364 | 0.0000 | 0.0000 | 0.9636 |

**Table S2** Vif between confounding factors

|  | GVIF | Df | GVIF^(1/(2*Df)) |
| --- | --- | --- | --- |
| class | 1.313887 | 5 | 1.027675 |
| marital_status | 1.197837 | 2 | 1.046163 |
| Ethnic_groups | 1.147771 | 2 | 1.035056 |
| CVD | 1.115519 | 1 | 1.056181 |
| Live_disease | 1.272335 | 1 | 1.127978 |
| age | 1.942820 | 1 | 1.393851 |
| charlson_score | 1.805141 | 1 | 1.343555 |
| APSIII | 1.260680 | 1 | 1.122800 |
| T | 1.182359 | 1 | 1.087363 |
| Hemoglobin | 1.108238 | 1 | 1.052729 |
| Neutrophils | 1.061955 | 1 | 1.030512 |
| PT | 1.132102 | 1 | 1.064003 |
| PTT | 1.107335 | 1 | 1.052300 |


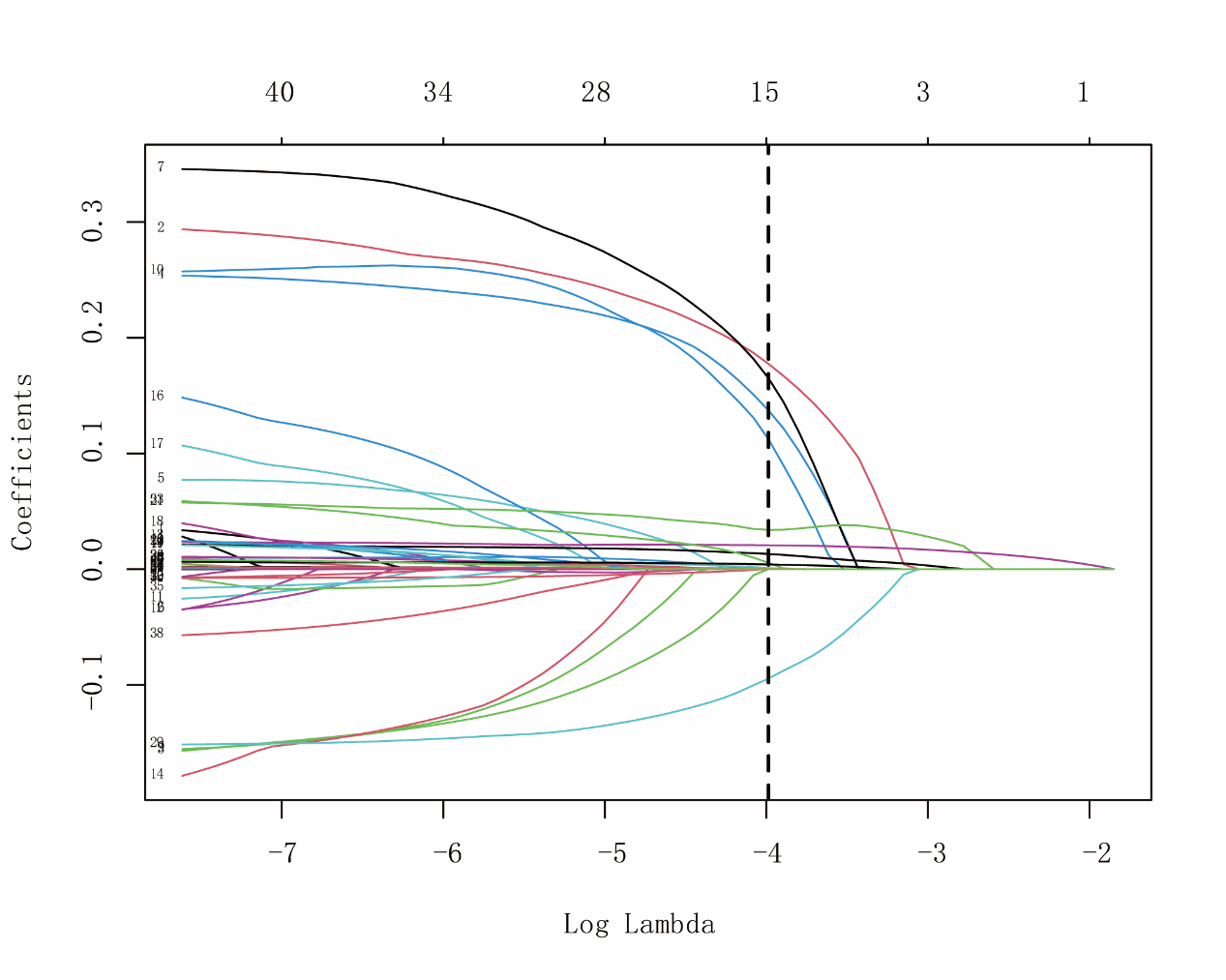


Figure S1: Coefficient path plot from LASSO regression.


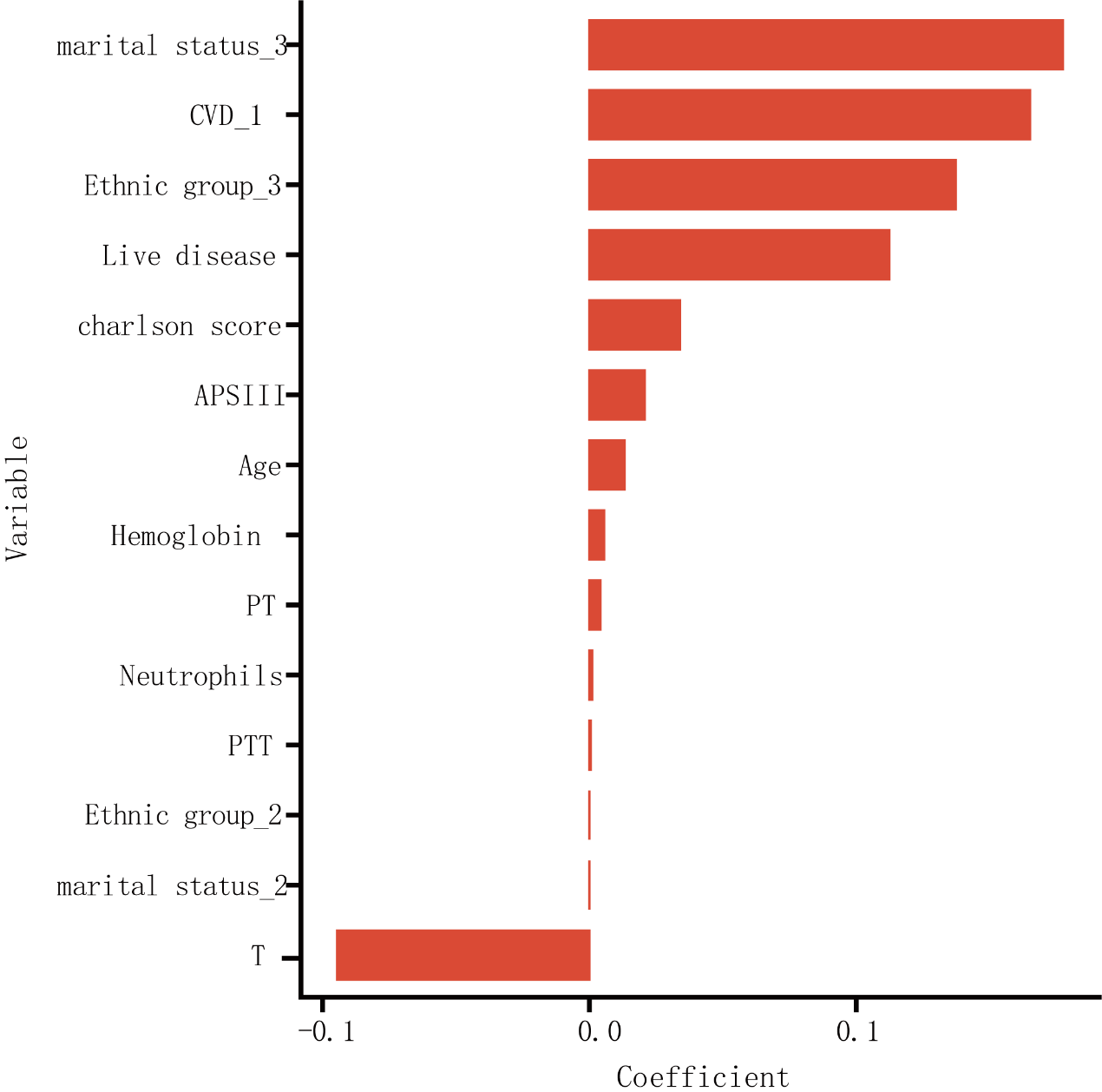


Figure S2: Coefficient bar plot from LASSO regression, where bar length and direction reflect each variable’s effect size and sign in the model.
